# Effects of Voluntary Exercise and Acetic Acid Supplementation on Skeletal Muscle Mitochondrial Function in Ovariectomized Mice

**DOI:** 10.3390/nu18020332

**Published:** 2026-01-20

**Authors:** Ki-Woong Park, Yoonhwan Kim, Yuan Tan, Byung-Jun Ryu, Seung-Min Lee, Hanall Lee, Byunghun So, Jinhan Park, Junho Jang, Chounghun Kang, Taewan Kim, Jinkyung Cho, Moon-Hyon Hwang, Jae-Geun Kim, Yong Kyung Kim, Young-Min Park

**Affiliations:** 1 Division of Health and Kinesiology, Incheon National University, Incheon 22012, Republic of Korea; kiwoongpark1995@gmail.com (K.-W.P.); tanyuan0312@gmail.com (Y.T.); zzbjryu@inu.ac.kr (B.-J.R.); seung3759@inu.ac.kr (S.-M.L.); mhwang@inu.ac.kr (M.-H.H.); 2 Department of Sport Science, Sungkyunkwan University, Suwon 16419, Republic of Korea; yh0524@skku.edu (Y.K.); tae.kim@skku.edu (T.K.); skkucjk81@skku.edu (J.C.); 3 Sport Science Institute & Health Promotion Center, Incheon National University, Incheon 22012, Republic of Korea; hlee2500@gmail.com; 4 Department of Health and Exercise Science, College of Graduate School, Inha University, Incheon 22212, Republic of Korea; sportshun@gmail.com (B.S.); sportsjinhan@gmail.com (J.P.); jangju2489@gmail.com (J.J.); ck@inha.ac.kr (C.K.); 5 Department of Physical Education, College of Education, Inha University, Incheon 22212, Republic of Korea; 6 Division of Life Sciences, College of Life Sciences and Bioengineering, Incheon National University, Incheon 22012, Republic of Korea; jgkim@inu.ac.kr; 7 Institute of Medical Science, College of Medicine, Yeungnam University, Daegu 38541, Republic of Korea

**Keywords:** ovariectomy, acetic acid, voluntary running, skeletal muscle, mitochondrial function

## Abstract

**Background:** Estrogen deficiency following human menopause or rodent ovariectomy (OVX) induces adverse alterations in body composition and metabolic function. This study investigated the combined effects of acetic acid supplementation and voluntary exercise on metabolic health and skeletal muscle mitochondrial function using an OVX mouse model. **Methods:** Forty female C57BL/6J mice (8 weeks old) were randomly assigned to 5 groups: sham (SHM), ovariectomized control (OVX), OVX with exercise (OVX-E), OVX with acetic acid (OVX-A), and OVX with both interventions (OVX-AE). Following a 1-week recovery from OVX, a 13-week intervention was initiated: 5% sodium acetate-supplemented chow and/or voluntary wheel running. Body composition, glucose tolerance, total energy expenditure, skeletal muscle mitochondrial function, and the contents of AMPKα, PGC-1α, and carbonyl protein were assessed. **Results:** OVX impaired whole-body metabolism and skeletal muscle mitochondrial function, specifically in the gastrocnemius muscle. While the exercise alone failed to mitigate the OVX-induced mitochondrial dysfunction, the combined treatment of exercise and acetic acid supplementation significantly rescued from the OVX-induced mitochondrial dysfunction. **Conclusions:** OVX resulted in detrimental changes in whole-body metabolism, but voluntary exercise and/or acetic acid supplementation had no rescuing effects on those parameters. In gastrocnemius muscle, acetic acid supplementation during exercise enhanced mitochondrial function in OVX mice.

## 1. Introduction

Ovarian dysfunction following human menopause and rodent ovariectomy results in a reduction of circulating estrogen level [1]. Estrogen deficiency is considered a key factor contributing to obesity in postmenopausal women [2]. The prevalence of obesity is more than three times higher in postmenopausal women than in premenopausal women, and according to the National Health and Nutrition Examination Survey (NHANES) in the United States, nearly two-thirds of women aged between 40 to 60 are overweight or obese [3]. These changes may result from the loss of estrogen’s regulatory effects on energy balance [4]. Supporting this, estrogen add-back protected against the ovariectomy (OVX)-induced increase in body weight and fat mass in rodent models [5]. Such menopause-associated alterations in body composition are associated with impaired metabolic health [6].

Mitochondria are essential organelles for metabolic health in eukaryotic cells [7]. However, menopause-induced obesity is identified as a major factor contributing to mitochondrial dysfunction and increased oxidative stress [8]. Excessive nutrient supply in adipose tissue promotes the production of reactive oxygen species (ROS) [9]. The accumulation of ROS induces oxidative stress, leading to structural damage and impaired cellular function [10]. Estrogen serves as a pivotal factor in sustaining mitochondrial structural integrity and suppressing ROS-mediated damage, thereby contributing to mitochondrial protection [11,12]. Notably, estrogen is closely linked to the activation of adenosine monophosphate-activated protein kinase alpha (AMPKα) [13] and peroxisome proliferator-activated receptor gamma coactivator-1α (PGC-1α) [14], which are key regulators of mitochondrial biogenesis and oxidative phosphorylation. This regulatory action can enhance the activation of mitochondrial respiratory complexes [15] and mitochondrial respiratory capacity [16]. The mitochondrial protective effects of estrogen diminish following menopause, leading to increased oxidative stress and mitochondrial dysfunction in skeletal muscle [17]. In OVXed mouse models, estrogen deficiency was shown to inhibit mitophagy and accelerate mitochondrial deterioration [18]. Collectively, mitochondrial dysfunction and ROS accumulation observed following menopause and OVX may result from the loss of estrogen-mediated mitochondrial protection.

Regular exercise is recognized as an effective strategy to improve mitochondrial function [19], contributing to improvements in body composition [20] and glucose tolerance [21] under estrogen-deficient conditions. Exercise-induced activation of AMPKα and PGC-1α plays a key role in improving mitochondrial function [22,23]. Voluntary wheel running led to increased expression of mitochondrial membrane proteins involved in oxidative phosphorylation and the electron transport chain in OVXed mouse models [24]. This positive effect on mitochondria is highly associated with counteracting oxidative stress such as ROS by increasing the expression of antioxidant enzymes in the skeletal muscle of OVXed mice [25].

Acetic acid is a major short-chain fatty acid produced through microbial fermentation of dietary carbohydrates in the gut, and can enter mitochondria in the form of acetyl-CoA, thereby contributing to ATP production [26]. Acetic acid has been shown to activate metabolic regulation through the phosphorylation of AMPKα in muscle cells [27]. Acetic acid supplementation improves fatty acid oxidation and metabolic parameters [28], and suppresses weight gain in mice with diet-induced obesity [29,30]. A dietary intervention using acetic acid-enriched chow showed increased levels of catabolic parameters including carnitine palmitoyltransferase 1B and phosphorylated AMPKα [31]. These metabolic benefits induced by acetic acid supplementation might be associated with enhanced skeletal muscle mitochondrial function and biogenesis [32]. To sum up, beneficial effects of exercise intervention or acetic acid supplementation alone on metabolic and mitochondrial health have been somewhat documented. However, whether acetic acid supplementation during exercise can provide a synergistic effect on skeletal muscle and whole-body metabolism is still unknown in estrogen-deficient conditions. Thus, the present study aimed to evaluate the combined effects of exercise intervention and acetic acid supplementation on whole-body and skeletal muscle metabolic health including mitochondrial function in an OVXed mouse model.

## 2. Methods

### 2.1. Animals

Forty female wild-type C57BL/6J mice (8 weeks old; C57BL/6J, DBL Co., Incheon, Republic of Korea) were randomly assigned to 5 experimental groups: (1) sham group (SHM, *n* = 8), (2) ovariectomized group (OVX, *n* = 8), (3) ovariectomized group with exercise intervention (OVX-E, *n* = 8), (4) ovariectomized group with acetic acid supplementation (OVX-A, *n* = 8), and (5) ovariectomized group with both acetic acid supplementation and exercise intervention (OVX-AE, *n* = 8). During the experimental period, each animal was single-housed, and environmental conditions were maintained at a constant temperature of 22 ± 1 °C and relative humidity of 50–80%, with a 12 h light/dark cycle. All procedures adhered to institutional guidelines and received approval from the Institutional Animal Care and Use Committee (INU-ANIM-2021-04).

### 2.2. Ovariectomy Surgeries

Following the acclimation period, all animals were anesthetized via inhalation of approximately 2% isoflurane (Hana Pharm. Co., Hwasung-si, Republic of Korea) using the respiratory delivery platform (Life Science Co., Irvine, CA, USA). Adequate anesthesia was confirmed by the absence of a withdrawal reflex in response to hindlimb stimulation. Once unresponsive, an eye gel containing carbomer (2 mg/g) was applied to the cornea to prevent dryness. OVX was performed while maintaining isoflurane at a concentration below 0.5%. The surgical site, located at the center of the dorsal surface, was shaved and sterilized before a midline incision (<0.5 cm). Using sterile forceps, the skin and underlying muscle layers were carefully separated, followed by an additional incision (<0.5 cm) on each side of the muscle layer. Both ovaries, along with the ovarian bursae and a portion of the oviducts, were excised. The incision sites were closed using small wound clips. In the SHM group, all surgical operations were identical except that the ovaries were not removed; their presence was instead confirmed visually. After surgeries, all animals were monitored twice daily during the recovery period to assess postoperative health and identify any abnormalities.

### 2.3. Acetic Acid and Voluntary Wheel Running

Dietary and exercise interventions were conducted for 13 weeks. The control diet groups (SHM, OVX, OVX-E) were provided with standard rodent chow (NIH-41KO; Zeigler Bros, Inc., Gardners, PA, USA), while the dietary intervention groups (OVX-A, OVX-AE) received the special chow supplemented with 5% (g/g) sodium acetate (S2889-1 kg, Sigma-Aldrich, Burlington, MA, USA) [29]. All animals had ad libitum access to food and water throughout the intervention. Food intake was weekly measured to calculating the amount consumed by weighing the remaining chow at a fixed time point.

For exercise intervention, mice in the OVX-E and OVX-AE groups were housed in cages equipped with a 10 cm-diameter running wheel to enable voluntary wheel running. The cages were standardized to dimensions of 13 cm × 23 cm × 14.5 cm. Mice had continuous access to the running wheel throughout the 13 weeks. The running distance was weekly monitored and calculated using a computerized counter.

### 2.4. Body Composition

Body weight was measured and recorded weekly throughout the experimental period. Total body lean and fat mass were assessed using dual energy X-ray absorptiometry (DXA, GE Medical Systems, Madison, WI, USA). To prepare for body composition analysis, food was withheld for 4 h before scanning. Each mouse underwent DXA scans 3 times, and the averaged values were used for data analysis. To minimize movement during the scanning procedure, animals were anesthetized via inhalation of isoflurane at a concentration of ~0.5%.

### 2.5. Metabolic Monitoring System

Total energy expenditure was assessed using an indirect calorimetry system (Promethion Metabolic Measurement System, Sable Systems, Las Vegas, NV, USA). Metabolic data were collected continuously over 7 days. Total energy expenditure was calculated based on oxygen consumption (VO_2_) and carbon dioxide production (VCO_2_), and data were analyzed using Expedata software (ver. 1.9.14, Sable Systems International, Las Vegas, NV, USA) [33]. The data were categorized into light and dark phases according to a 12 h light/dark cycle. Throughout the measurement period, mice had unrestricted access to food and water. The averaged values from the 5 days, excluding the first day (acclimation) and the last day, were used for data analysis.

### 2.6. Glucose Tolerance Test

Mice were fasted for 12 h before testing. A 20% sterile glucose solution (g/mL, glucose/distilled water) was administered via intraperitoneal injection (IP) at a dose of 2 g/kg body weight. Blood samples were collected from the tail vein, and blood glucose levels were measured by the glucometer (Accu-chek Performa, Roche Diagnostics, Mannheim, Germany) at 6 time points: pre-injection (0 min) and at 15, 30, 45, 60, and 120 min post-injection. At each time point, 5 to 10 µL of blood was used for glucose measurement. Glucose tolerance was evaluated by calculating the area under the curve (AUC). AUC was determined by integrating the blood glucose curve from 0 to 120 min after the glucose injection [33].

### 2.7. Mitochondrial Function

Mitochondrial function was assessed using a modified protocol based on previously established methods [34]. Measurement of skeletal muscle mitochondrial respiration was conducted at the end of the 13-week intervention period, concurrently with tissue collection. Mice were fasted for 12 h before tissue harvesting. Anesthesia was induced via intraperitoneal injection of 2.5% (g/mL) tribromoethanol solution (2,2,2-tribromoethanol; T48402-25G, Sigma-Aldrich, Burlington, MA, USA) at a dosage of 0.01 mL/g body weight. Following anesthesia, the gastrocnemius and soleus muscles were excised, weighed immediately, and used for mitochondrial respiration analysis. Upon completion of tissue preparation, muscle specimens were incubated in 2 mL MiR05 (0.5 mM EGTA, 3 mM MgCl_2_·6H_2_O, 60 mM K-lactobionate, 20 mM taurine, 10 mM KH_2_PO_4_, 20 mM HEPES, 110 mM sucrose, 1 g/L fatty acid-free bovine serum albumin (BSA)) using the Oxygraph O_2_k chamber (Oroboros Instruments, Innsbruck, Austria). Oxygen concentration within the chamber was adjusted to 350–380 nmol/mL using catalase and hydrogen peroxide. Once the oxygen signal stabilized, substrates were sequentially injected using a precision Hamilton syringe: pyruvate (5 mM) and malate (0.5 mM), adenosine diphosphate (ADP, 2 mM), and succinate (9 mM). The analysis of mitochondrial respiratory function was performed through comparison of oxygen consumption rates across substrate stages using DatLab 7.4 software. Results were expressed as oxygen flux [pmol/(s·mg)] normalized to tissue weight.

### 2.8. Western Blot Analysis (AMPKα, PGC-1α, and Carbonyl Protein)

Western blot analysis was conducted based on the previously established protocol [35]. During tissue harvesting, the gastrocnemius and soleus muscles were weighed and then immediately snap-frozen in liquid nitrogen. Frozen tissues were homogenized using a FastPrep-24™ 5 G bead homogenizer (MP Biomedicals, Irvine, CA, USA) in RIPA buffer (Cell Nest, Hanam-si, Republic of Korea) containing protease and phosphatase inhibitors (GenDEPOT, Seoul, Republic of Korea). Protein concentrations were quantified using the Pierce BCA protein assay kit (Thermo Fisher Scientific, Waltham, MA, USA). Homogenized samples were heated at 95–100 °C for approximately 5 min, followed by centrifugation to remove impurities. The proteins were separated via SDS-PAGE using a Criterion vertical gel electrophoresis system (Bio-Rad, Hercules, CA, USA), and subsequently transferred to PVDF membranes (Amersham, Freiburg, Germany) using a Criterion blotter wet transfer system (Bio-Rad, Hercules, CA, USA). Membranes were blocked in TBS-T buffer (Tris-buffered saline with Tween 20) containing 5% BSA. The membranes were incubated with primary antibodies at 4 °C for ~12 h (Table 1), followed by incubation with secondary antibodies at room temperature for 1 h. Protein carbonylation, a marker of oxidative stress, was measured using a carbonyl assay kit (ab178020, Abcam, Waltham, MA, USA) in combination with 2,4-dinitrophenylhydrazine (DNPH). Detection was performed using ECL Western blotting substrate (Thermo Fisher Scientific, Waltham, MA, USA). Quantification of protein content was performed using densitometric measurement (Bio-Rad, Hercules, CA, USA). The resulting images were analyzed using ImageJ software (version 1.8.0_172, NIH, Bethesda, MD, USA).

### 2.9. Statistical Analysis

To analyze group differences in weekly energy intake and voluntary wheel running distance during the intervention, a repeated measures ANOVA (group × time) was conducted. One-way ANOVA was used to compare group differences in body weight and composition, glucose tolerance, mitochondrial function, and protein contents (i.e., AMPKα, PGC-1α, and carbonylated protein). ANCOVA with body mass as a covariate was used to compare group differences in total energy expenditure. Upon detection of a significant main effect, Fisher Least Significant Difference test was conducted as a post hoc analysis. Effect sizes were calculated to support interpretation of the magnitude of group differences (eta-squared, η^2^, for one-way ANOVA). All statistical analyses were conducted using the IBM SPSS Statistics for Windows (version 25.0; IBM Corp., Armonk, NY, USA), and statistical significance was set at *p* < 0.05. All of the data are reported as mean ± standard error (SE).

## 3. Results

### 3.1. Body Composition

**Figure 1:**
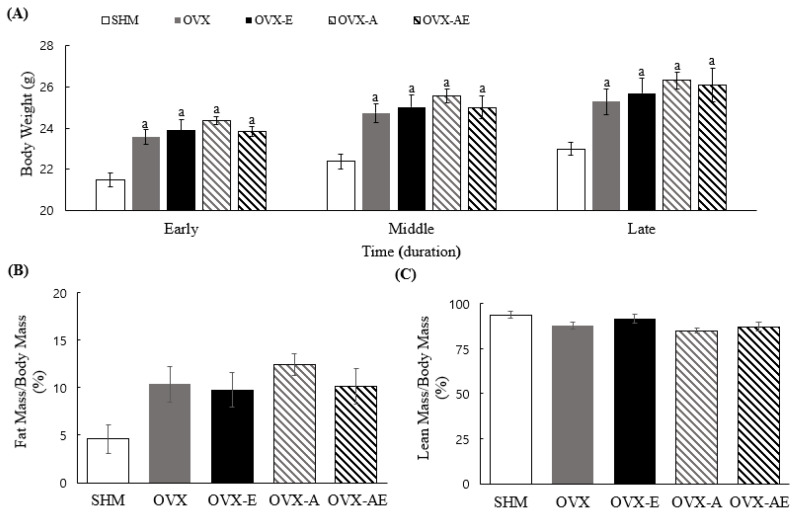
Body composition. (**A**) Differences of body weight between groups per period, (**B**) differences of fat mass between groups, and (**C**) differences of lean mass between groups. Values are means ± SE. Animal *n* = 7–8/group. ^a^ a significant difference compared to the SHM. SHM: sham group; OVX: ovariectomy group; OVX-E: ovariectomy + exercise; OVX-A: ovariectomy + acetic acid; OVX-AE: ovariectomy + acetic acid + exercise; Early phase: weeks 1–4; Middle phase: weeks 5–9; and Late phase: weeks 10–13.

Significant differences in body weight were observed between groups during all phases: early (weeks 1–4), middle (weeks 5–9), and late (weeks 10–13) phases (η^2^ = 0.567, *p* < 0.001; η^2^ = 0.494, *p* < 0.001; and η^2^ = 0.405, *p* = 0.002, respectively; Figure 1A). In all phases, the SHM group exhibited significantly lower body weight compared to other groups (all, *p* < 0.001). The SHM group showed a trend towards lowering the ratio of fat to body mass than OVX, OVX-E, OVX-A, and OVX-AE groups (main group effect, η^2^ = 0.248, *p* = 0.052; Figure 1B). There were no significant differences between groups on the ratio of lean to body mass (Figure 1C).

### 3.2. Wheel Running Distance and Food Intake

During the 13-week intervention, there was no significant main effect or interaction on voluntary wheel running distance, while the significant main effect for both time and group was found on food intake (all, *p* < 0.001). Food intake showed a decreasing trend over time (Week 1 vs. Week 13: 611.606 ± 16.680 vs. 497.736 ± 14.443 kcal/kg; η^2^ = 0.378, *p* < 0.001). The SHM group exhibited significantly higher food intake as compared with other groups (all, *p* < 0.001), with no significant differences between all OVX groups.

### 3.3. Total Energy Expenditure

**Figure 2:**
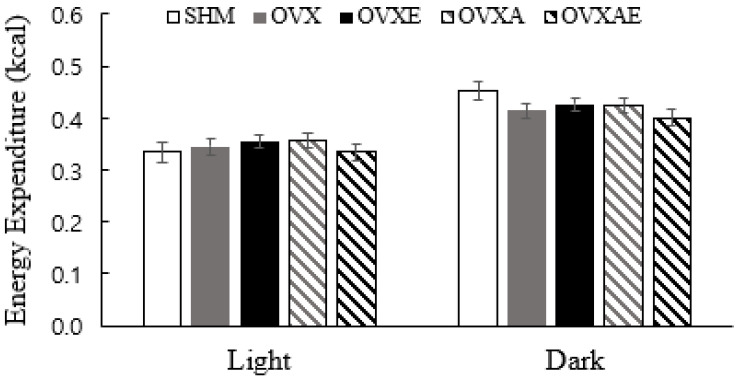
Total Energy Expenditure. Total energy expenditure during each cycle. Values are estimated means ± SE (ANCOVA with total mass as a covariate). Animal *n* = 6–7/group. SHM: sham group; OVX: ovariectomy group; OVX-E: ovariectomy + exercise; OVX-A: ovariectomy + acetic acid; OVX-AE: ovariectomy + acetic acid + exercise; Light: inactivity time; Dark: activity time.

The analysis showed no significant differences between groups in the average daily total energy expenditure during the light (inactive) and dark (active) cycle. During the dark cycle, the SHM group showed a trend to be greater than OVX and OVX-AE groups (*p* = 0.090 and *p* = 0.052, respectively; Figure 2).

### 3.4. Glucose Tolerance Test

**Figure 3:**
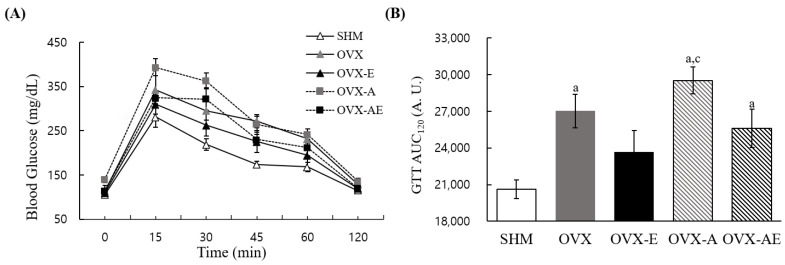
Glucose tolerance. (**A**) Circulating glucose kinetics and (**B**) area under the curve during the test. Values are means ± SE. Animal *n* = 6–7/group. ^a^ a significant difference in comparison with the SHM group; ^c^ a significant difference in comparison with the OVX-E group. SHM: sham group; OVX: ovariectomy group; OVXE: ovariectomy + exercise; OVX-A: ovariectomy + acetic acid; OVX-AE: ovariectomy + acetic acid + exercise; GTT AUC_120_: area under the curve during the test.

Intraperitoneal glucose tolerance test revealed significant group differences in glucose area under the curve (AUC), an index of glucose uptake resistance (η^2^ = 0.434, *p* < 0.001; Figure 3B). The SHM group exhibited significantly lower glucose AUC as compared with OVX, OVX-A, and OVX-AE groups (*p* = 0.002; *p* < 0.001; and *p* = 0.017, respectively). The OVX-E group found significantly lower glucose AUC compared to OVX-A group (*p* = 0.003).

### 3.5. Mitochondrial Function in Skeletal Muscle

**Figure 4:**
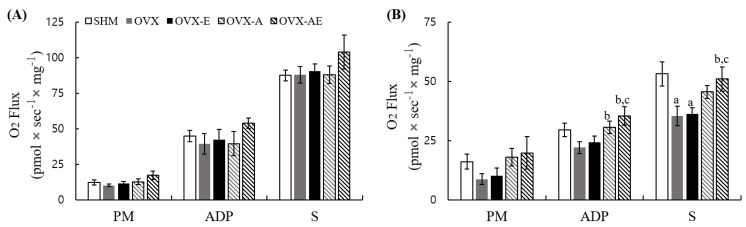
Mitochondrial function. Oxygen consumption rates in skeletal muscle (**A**) soleus and (**B**) gastrocnemius. Values are means ± SE. Animal *n* = 6–7/group. ^a^ a significant difference in comparison with the SHM group; ^b^ a significant difference in comparison with the OVX group; ^c^ a significant difference in comparison with the OVX-E group. SHM: sham group; OVX: ovariectomy group; OVXE: ovariectomy + exercise; OVX-A: ovariectomy + acetic acid; OVX-AE: ovariectomy + acetic acid + exercise; PM: pyruvate + malate; ADP: adenosine diphosphate; S: succinate.

Mitochondrial function was assessed by measuring oxygen consumption rates. In the soleus muscle, the analysis showed no significant group differences in oxygen consumption rates across all substrate conditions (PM, ADP, and S; Figure 4A). In the gastrocnemius muscle, significant group differences were observed in the ADP and S, but not in the PM condition (PM, η^2^ = 0.192, *p* = 0.202; ADP, η^2^ = 0.336, *p* = 0.026; and S, η^2^ = 0.379, *p* = 0.010, respectively; Figure 4B). During the ADP condition, oxygen consumption rate was significantly lower in the OVX compared to the OVX-A and OVX-AE groups (*p* = 0.035 and *p* = 0.004, respectively). Oxygen consumption rate was significantly lower in the OVX-E compared to the OVX-AE group (*p* = 0.015). In the succinate (S) condition, the SHM group exhibited significantly higher oxygen consumption rate as compared with the OVX and OVX-E groups (*p* = 0.003 and *p* = 0.006, respectively). The OVX-AE group also showed significantly higher oxygen consumption rate as compared with the OVX and OVX-E groups (*p* = 0.015 and *p* = 0.025, respectively).

### 3.6. Protein Contents in Skeletal Muscle

**Figure 5:**
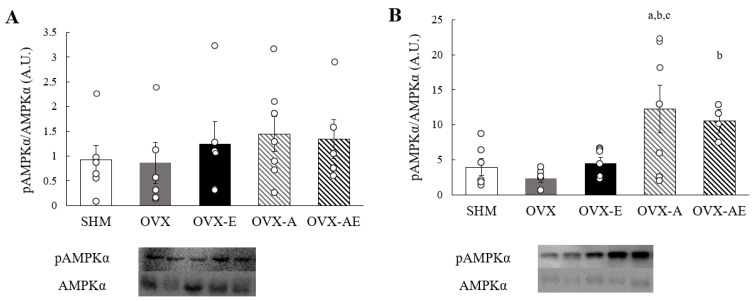
AMPKα protein contents. (**A**) the ratio of phosphorylated AMPKα to AMPKα in soleus, and (**B**) the ratio of phosphorylated AMPKα to AMPKα in gastrocnemius. Values are means ± SE. Sample *n* = 4–7/group. ^a^ a significant difference compared to the SHM group; ^b^ a significant difference compared to the OVX group; ^c^ a significant difference in comparison with the OVX-E group. SHM: sham group; OVX: ovariectomy group; OVX-E: ovariectomy + exercise; OVX-A: ovariectomy + acetic acid; OVX-AE: ovariectomy + acetic acid + exercise.

On both soleus and gastrocnemius muscles, the analysis showed no significant group differences in AMPKα and phosphorylated AMPKα (pAMPKα) protein contents. Whereas no significant differences were found in the ratio of pAMPKα to AMPKα in the soleus muscle (Figure 5A), a significant main group effect was observed for this ratio in the gastrocnemius muscle (η^2^ = 0.425, *p* = 0.013; Figure 5B). A significantly elevated pAMPKα/AMPKα ratio was observed in the OVX-A group relative to the SHM, OVX, and OVX-E groups (*p* = 0.008, *p* = 0.003, and *p* = 0.013, respectively). The OVX-AE group showed a significantly greater ratio as compared with the OVX group (*p* = 0.04).

The PGC-1α and carbonyl protein contents showed no significant differences between groups in the soleus and gastrocnemius muscles.

## 4. Discussion

The present study aimed to evaluate the combined effects of exercise and acetic acid supplementation on whole-body and skeletal muscle metabolic health in an OVXed mouse model. OVX resulted in detrimental changes in whole-body metabolism (body weight and GTT), with a trend towards increasing fat mass and decreasing whole-body metabolic function. There were no rescuing effects of exercise and/or acetic acid supplementation on the parameters of whole-body metabolism. Interestingly, in the gastrocnemius but not soleus muscle, OVX significantly decreased mitochondrial function. However, acetic acid supplementation during exercise protected against the OVX-induced mitochondrial dysfunction, while the exercise intervention alone failed to mitigate the mitochondrial dysfunction. Furthermore, our results showed that acetic acid supplementation enhanced AMPKα phosphorylation.

Estrogen is indispensable for regulating body composition and metabolic function [36]. Previous study indicated that OVX led to unfavorable physiological changes, such as increased adiposity and impaired metabolic function [37]. Indeed, various experimental studies using OVX mouse models consistently demonstrated detrimental alterations in body composition and metabolic efficiency as compared with sham-operated controls [38]. Clinical study also showed that surgical removal of the ovaries is associated with increased body weight and fat mass [39] and impaired glucose tolerance [40]. With this body of evidence, the current study confirmed that the OVX exhibited detrimental effects in body weight and glucose tolerance, along with a trend towards increasing fat mass and lowering total energy expenditure, as compared with the SHM group. These findings can be attributed to the reduction in circulating estrogen levels following OVX, which likely led to the loss of estrogen’s regulatory roles in maintaining metabolic homeostasis and healthy body composition.

In the current study, neither exercise intervention nor acetic acid supplementation effectively mitigated the increases in body weight and fat mass induced by OVX. This lack of intervention effect may be attributed to the insufficient exercise volume acquired from the voluntary wheel running. Previous evidence showed that female C57BL/6J mice typically run an average of approximately 10 km per day under voluntary wheel running conditions [41,42]. In the current study, OVX-E and OVX-AE groups achieved considerably lower running distances, averaging only 0.77 ± 0.48 km/day and 0.68 ± 0.20 km/day, respectively. Consistent with the findings of the current study, previous studies also showed that OVX mice reported a 63% reduction in voluntary wheel running distance as compared with controls. Another previous study showed that OVX Wistar rats exhibited over an eight-fold reduction in running distance relative to sham-operated rats [43]. These data suggest that OVX-induced estrogen deficiency may be a major contributing factor to the reduction in voluntary physical activity levels. A previous study is consistent with our findings, in that no significant difference in body weight was found between OVX mice and those subjected to voluntary wheel running (32.7 ± 0.27 g vs. 33.5 ± 0.31 g) [44]. Taken together, these results highlight that establishing a minimum threshold of physical activity volume would be required to induce improvements in body composition of the OVX rodent model. Future studies should focus on defining the effective exercise volume in menopausal models.

In addition to exercise intervention, acetic acid supplementation also failed to produce significant improvements in body weight and composition in the OVX mice in the current study. The result contrasts with findings from a previous study, which showed that daily ingestion of vinegar-based acetic acid for 12 weeks led to dose-dependent reductions in body weight and fat mass in overweight adults (BMI 25–30 kg/m^2^), while the placebo group exhibited either weight maintenance or gain [45]. The discrepancy between those studies may be partially due to species-specific differences in acetic acid metabolism between humans and rodents. A comprehensive review on the metabolic effects of acetic acid suggested that, although orally ingested or microbially produced acetic acid in the intestine exerts favorable metabolic effects in humans, it may paradoxically promote obesity and insulin resistance in certain rodent models [46]. Indeed, a previous finding using rodents reported that high levels of acetic acid supplementation stimulated the parasympathetic nervous system, enhancing the secretion of appetite-stimulating hormones such as ghrelin, gastrin, and insulin in response to glucose, and ultimately increasing energy intake and storage [47]. In contrast, a human study showed that acetic acid supplementation may act on the hypothalamus to promote appetite-suppressing signals [48]. Taken together, these findings underline that the results of acetic acid supplementation on body composition are somewhat mixed, possibly due to the discrepancy of species and experimental conditions (OVX, obesity, supplementing dose, etc.).

According to the results of the current study, the average mitochondrial respiration measured across all experimental groups was significantly higher in the soleus as compared with the gastrocnemius muscle (soleus vs. gastrocnemius, 90.86 ± 2.38 vs. 44.02 ± 1.73 pmol·s^−1^·mg^−1^, respectively). This disparity may be attributed to differences in muscle fiber type composition between those muscles because skeletal muscles exhibit distinct fiber-type distributions depending on their anatomical location [49]. The soleus muscle is proximal and predominantly composed of oxidative type I and type IIa fibers, which consist of up to 90% of the total fibers, whereas the gastrocnemius contains a higher proportion (up to 60%) of glycolytic type IIx fibers [50]. Notably, type I fibers are known to exhibit higher activity levels of key antioxidant enzymes, such as superoxide dismutase, catalase, and glutathione peroxidase, as compared with type IIx fibers [51]. Not surprisingly, the soleus muscle consists mainly of oxidative and mitochondria-rich fibers with superior antioxidant defenses, demonstrating better mitochondrial function than the gastrocnemius [52].

In the current study, significant group differences in mitochondrial function were observed only in the gastrocnemius, but not in the soleus muscle. Whereas the soleus showed no changes following OVX or other treatments, the gastrocnemius exhibited OVX-induced mitochondrial dysfunction, which was rescued by the combined treatment of exercise and acetic acid supplementation. Indeed, a prior animal study supports the current study in that the soleus mitochondrial function showed no response to the hindlimb unloading and sirtuin activator (SRT2104) administration, whereas the gastrocnemius displayed significant changes in mitochondrial function and drug efficacy following those treatments [53]. The underlying mechanisms of the differential sensitivity to intervention between the soleus and gastrocnemius muscles might be partially related to different compositions of muscle fiber types, which possess different contents of antioxidant enzymes [51]. Whether the greater capacity of antioxidant enzymes in the soleus muscle can protect against the OVX-induced mitochondrial dysfunction should be further studied in other various pre-clinical models.

In the gastrocnemius, the current study demonstrated that mitochondrial function was significantly lower in the OVX group than the SHM group (53.05 ± 5.03 vs. 35.31 ± 4.13 pmol·s^−1^·mg^−1^, respectively). In line with the results of the current study, a previous study reported that OVX in C57BL/6J mice led to impaired mitochondrial function in skeletal muscle as compared with sham-operated controls [54]. Estrogen deficiency induced by OVX can directly negatively contribute to the mitochondrial function in skeletal muscle [55]. Also, reduced ATP production and downregulated expression of oxidative phosphorylation-related proteins are associated with mitochondrial dysfunction in menopausal models [56]. Additionally, OVX-induced increases in body weight and fat mass may indirectly contribute to mitochondrial impairment. The accumulation of adipose tissue promotes the generation of ROS, which disrupts key signaling pathways involved in mitochondrial metabolism [57]. An obese mouse model found elevated expression and activation of the GTP-binding protein RalA, persistent activation of which can induce the dysregulation of mitochondrial fission, ultimately leading to functional decline [58].

In the gastrocnemius muscle, the analysis of mitochondrial function revealed that exercise intervention alone did not improve the OVX-induced decline in mitochondrial function, contrasting with previous findings reporting beneficial effects of exercise in the menopausal model [59]. Presumably, compromised metabolism in OVX mice with reduced levels of spontaneous physical activity may contribute to the negative response to exercise intervention. In contrast, acetic acid supplementation alone partially attenuated the OVX-induced mitochondrial dysfunction (*p* = 0.075). Notably, acetic acid supplementation with exercise significantly rescued from the OVX-induced mitochondrial dysfunction, implying a strong effect of combined treatments. Molecular analysis further revealed that acetic acid supplementation increased the phosphorylated levels of AMPKα, a key regulator of mitochondrial function. Acetic acid is known to act as a metabolic signaling molecule that induces AMPKα phosphorylation, thereby promoting glucose utilization and fatty acid oxidation in skeletal muscle [27]. A previous study using Otsuka Long-Evans obese rats demonstrated that oral administration of acetic acid enhanced AMPKα phosphorylation, along with the increase in mitochondrial oxygen consumption and lipid oxidation capacity [60]. Moreover, previous findings showed that acetic acid supplementation increased key mitochondrial metabolites in skeletal muscle, including plasma acetate, acetyl-CoA, and acetylcarnitine, which can be favorably used as energy during exercise [61]. These findings suggest that acetic acid supplementation may provide the enhancement of mitochondrial metabolites and AMPKα activation, ultimately supporting mitochondrial oxygen consumption and function.

Despite the significant increase in AMPKα phosphorylation observed in the current study, no group differences were detected in the protein contents of PGC-1α, a well-known downstream effector of AMPKα. This may suggest that AMPKα activation does not necessarily lead to an upregulation of PGC-1α expression. Gurd et al. [62] reported that the relationship between AMPKα activation and PGC-1α expression was inconsistent under various exercise conditions. Another study also showed that treatment with metformin, a canonical AMPKα activator, increased AMPKα phosphorylation without changes in PGC-1α expression [63]. To sum up, while AMPKα and PGC-1α often act in concert, their regulations may be uncoupled under certain physiological conditions or stimuli. Additionally, although some previous studies reported an increase in protein carbonylation, a marker of oxidative stress, following OVX [64,65], no significant differences between groups were observed in the current study.

The current study has several limitations. First, this study used 8-week-old female wild-type C57BL/6J mice and employed OVX surgery to model human menopause. While this surgical model is widely used as a gold-standard for inducing sex hormone deficiency, it may differ physiologically from natural menopause, which is more likely related to chronological aging. Nevertheless, using young mice in the current study allowed us to minimize the potential confounding effects of age-related metabolic disorders and to investigate more precisely the metabolic consequences of gonadal dysfunction. Second, Voluntary wheel running was employed in this current study as a model of exercise intervention. Whereas this intervention approach is less stressful for experimental animals, it cannot provide standardized exercise volume across animals because animals have free access to the running wheels. High variability in exercise volume possibly disguised the true effects of exercise in some outcomes of the current study. Future studies should consider employing a treadmill-based forced exercise protocol to standardize exercise intensity and volume. Third, both food and water were provided ad libitum during the intervention, and acetic acid was administered by mixing it into the normal chow with a certain composition. Individual differences in the volume of food intake can contribute to the dose of the acetic acid supplementation. Moreover, orally consumed acetic acids undergo gastrointestinal digestion and may provide physiological effects distinct from the effects of endogenously produced acetic acid generated by gut microbiota fermentation. The different methodological approaches of the acetic acid treatment may alter the results of the current study; thus, careful interpretation of the results is required. Fourth, the current study did not include sham groups with the same interventions as the OVX group. Future study is necessary to investigate whether the effects of acetic acids and exercise are altered in SHM compared to OVX groups. Fifth, the current study did not directly measure plasma or intestinal concentrations of acetic acids following supplementation, limiting the ability to confirm whether acetic acid exerted direct effects on muscle metabolism. Lastly, given the exploratory nature of this preclinical study and the ethical constraints on animal numbers, no formal a priori sample size calculation was performed. As the study may be underpowered to detect small effects between groups, non-significant findings should be cautiously interpreted.

## 5. Conclusions

The current study demonstrated that OVX induced impairments in whole-body metabolism and gastrocnemius muscle mitochondrial function. In the gastrocnemius muscle, exercise alone failed to attenuate the OVX-induced mitochondrial dysfunction. However, acetic acid supplementation during exercise rescued the OVX-induced mitochondrial dysfunction. Acetic acid supplementation enhanced AMPKα phosphorylation, which might be associated with increased mitochondrial function. Additional research is necessary to investigate (1) the underlying mechanism of no rescuing effect of exercise in OVX mice, and (2) the optimal exercise intensity and volume to rescue the OVX-induced metabolic dysfunction in whole body and skeletal muscle.

## Figures and Tables

**Table 1:** List of primary antibodies.

Primary Antibody	Host	Clonality	Source (Catalog No.)	Dilution
Anti-AMPKα	Rabbit	Polyclonal	Cell Signaling (Danvers, MA, USA) (#2532)	1:2000
Anti-pAMPKα (Thr172)	Rabbit	Monoclonal	Cell Signaling (Danvers, MA, USA) (#2535)	1:2000
Anti-PGC-1α	Mouse	Monoclonal	Santa Cruz Biotech (Dallas, TX, USA) (#sc-517380)	1:125
Anti-GAPDH	Rabbit	Polyclonal	Enogene Biotech (Rockville, MD, USA) (#E1C604)	1:2000

## Data Availability

The original contributions presented in this study are included in the article/Supplementary Materials. Further inquiries can be directed to the corresponding authors.

## Supplementary Materials

The following supporting information can be downloaded at: https://www.mdpi.com/article/10.3390/nu18020332/s1.
